# Impact of therapeutic plasmapheresis on the duration of organ failure in patients with hypertriglyceridemia-associated acute pancreatitis

**DOI:** 10.1186/s13613-024-01285-3

**Published:** 2024-04-15

**Authors:** Lanting Wang, Jing Zhou, Cheng Lv, Donghuang Hong, Zuozheng Wang, Wenjian Mao, Yuxiu Liu, Zixiong Zhang, Yuanzhen Li, Gang Li, Bo Ye, Baiqiang Li, Longxiang Cao, Zhihui Tong, Weiqin Li, Lu Ke, for the Chinese Acute Pancreatitis Clinical Trials Group (CAPCTG), Qi Yang, Jiajia Lin, Lin Gao, Yan Chen, Nonghua Lv, Yin Zhu, Liang Xia, Wenhua He, Zhenping Chen, Xinting Pan, Qingyun Zhu, Youdong Wan, Hong Mei, Kang Li, Miao Chen, Chengjian He, Hongyi Yao, Zigui Zhu, Weili Lu, Weihua Gu, Feng Zhou, Shumin Tu, Long Fu, Bing Xue, Haibin Ni, Xiaofei Huang, Dandan Zhou, Guoxiu Zhang, Lening Ren, Dahuan Li, Xiangyang Zhao, Wei Zhao, Xiaomei Chen, Junli Sun, Keke Xin, Weiwei Chen, Qingcheng Xu, Jingchun Song, Qingbo Zeng, Min Shao, Dongsheng Zhao, Jianfeng Tu, Hongguo Yang, Bin Wu, Huaguang Ye, Mingzhi Chen, Mei Yang, Hong Gao, Qiang Li, Lijuan Zhao, Guobing Chen, Yafei Li, Honghai Xia, Dongliang Yang, Shusheng Zhou, Siyao Liu, Jiyan Lin, Songjing Shi, Weijie Yao, Shan Xu, Lei Yu, Feng Guo, Yongjun Lin, Yun Zhou, Yue Long, Guixian Luo, Quanxing Feng, Zhiyong Liu

**Affiliations:** 1 Department of Critical Care Medicine, Jinling Hospital, Affiliated Hospital of Medical School, Nanjing University, No. 305 East Zhongshan Road, Nanjing, 210002 China; 2 Research Institute of Critical Care Medicine and Emergency Rescue at Nanjing University, Nanjing, China; 3 Department of Critical Care Medicine, Jinling Hospital, Nanjing Medical University, Nanjing, China; 4 Department of Critical Care Medicine, Fujian Provincial Hospital, Fuzhou, China; 5 Department of Hepatobiliary Surgery, General Hospital of Ningxia Medical University, Yinchuan, China; 6 National Institute of Healthcare Data Science, Nanjing University, Nanjing, China; 7 Department of Biostatistics, School of Public Health, Southern Medical University, Guangzhou, China

**Keywords:** Acute pancreatitis, Hypertriglyceridemia, Organ failure, Plasmapheresis

## Abstract

**Background:**

Plasmapheresis is widely used for severe hypertriglyceridemia-associated acute pancreatitis (HTG-AP) to remove excessive triglycerides from plasma. This study aimed to evaluate whether plasmapheresis could improve the duration of organ failure in HTG-AP patients.

**Methods:**

We analyzed a cohort of patients from a multicenter, prospective, long-running registry (the PERFORM) collecting HTG-AP patients admitted to the study sites within 72 h from the onset of symptoms. This study was based on data collected from November 2020 to March 2023. Patients who had organ failure at enrollment were involved in the analyses. The primary outcome was time to organ failure resolution within 14 days. Multivariable Cox regression model was used to evaluate the association between plasmapheresis and time to organ failure resolution. Directed acyclic graph (DAG) was used to identify potential confounders.

**Results:**

A total of 122 HTG-AP patients were included (median [IQR] sequential organ failure assessment (SOFA) score at enrollment, 3.00 [2.00–4.00]). Among the study patients, 46 underwent plasmapheresis, and 76 received medical treatment. The DAG revealed that baseline serum triglyceride, APACHE II score, respiratory failure, cardiovascular failure, and renal failure were potential confounders. After adjusting for the selected confounders, there was no significant difference in time to organ failure resolution between patients undergoing plasmapheresis and those receiving exclusive medical treatment (HR = 1.07; 95%CI 0.68–1.68; P = 0.777). Moreover, the use of plasmapheresis was associated with higher ICU requirements (97.8% [45/46] vs. 65.8% [50/76]; OR, 19.33; 95%CI 2.20 to 169.81; P = 0.008).

**Conclusions:**

In HTG-AP patients with early organ failure, plasmapheresis was not associated with accelerated organ failure resolution compared to medical treatment but may be associated with more ICU admissions.

*Trial registration*: The PERFORM study was registered in the Chinese Clinical Trial Registry (ChiCTR2000039541). Registered 30 October 2020.

**Supplementary Information:**

The online version contains supplementary material available at 10.1186/s13613-024-01285-3.

## Introduction

The prevalence of hypertriglyceridemia-associated acute pancreatitis (HTG-AP) is increasing worldwide, especially in China [1–3], which has been attributed to the rapidly changing lifestyle [4, 5] and genetic background [6] of the Chinese population. Compared to other etiologies, HTG-AP patients are more likely to develop organ failure (OF) [7, 8], which is significantly associated with higher mortality when it persists longer [9, 10].

Previous studies found that elevated serum triglyceride level was dose-dependently correlated with the incidence of persistent OF [11, 12], therefore efforts had been made to lower serum triglyceride during the early phase of HTG-AP. Lu et al. found that timely lowering serum triglyceride to less than 5.65 mmol/L during the early phase of HTG-AP was associated with improved OF [13]. Thus, therapies aiming at rapid triglyceride decline may improve clinical outcomes.

Plasmapheresis, which could theoretically remove triglyceride and inflammatory cytokines from plasma efficiently [14], is widely applied in the management of HTG-AP. Many retrospective studies [15–17] demonstrated a more significant reduction of triglyceride with plasmapheresis than medical triglyceride-lowering therapies. However, none of the studies assessed whether plasmapheresis could shorten the duration of organ failure. Our previous study [18] showed that plasmapheresis was not associated with the incidence and duration of organ failure in a cohort of HTG-AP patients. However, only 30% (81/267) patients had organ failure at enrollment, and 42.3% (113/267) study patients were mild cases.

In this study, we aimed to evaluate whether the use of plasmapheresis was associated with accelerated organ failure resolution in more severe HTG-AP patients who had organ failure before treatment. The number and type of organ failure were additionally considered.

## Methods

### Study design

This is a multicenter, prospective cohort study using data collected between November 2020 and March 2023 from the PERFORM study registry (Chinese Clinical Trial Registry, ChiCTR2000039541), which prospectively recruited HTG-AP patients in the participating sites across China. All ethics committees of the study sites approved the study, and written informed consent was obtained from all participants or next of kin before enrollment.

### Study population

In the PERFORM study, acute pancreatitis patients aged 18–70 years admitted to any of the participating sites were consecutively screened. Patients within 72 h from the onset of abdominal pain, with serum triglyceride level > 11.3 mmol/L on admission, and accompanied by at least one of the worrisome features were enrolled. The definition of worrisome features was provided in the protocol [19]. Exclusion criteria were (1) pregnant or lactating, (2) failure to obtain informed consent, and (3) expected to die within 48 h after enrollment. In the present study, we additionally excluded the patients who did not have any OF at enrollment, received blood purification therapy other than plasmapheresis after enrollment, did not initiate plasmapheresis within two days of enrollment, and were without complete data for analyses. Organ failure was defined as an organ-specific individual Sequential Organ Failure Assessment (SOFA) score of two or more for the respiratory, cardiovascular, or renal system. Patients who received plasmapheresis therapy were categorized into the plasmapheresis group, and those who did not were categorized into the medical group.

### Clinical management

All patients received standard medical treatment, including intravenous fluids, nutrition prescriptions, analgesics, and organ support when appropriate. For patients who received plasmapheresis therapy, the detailed prescriptions of plasmapheresis (vascular access, total plasma volume, number of sessions, duration of one session, anticoagulation, and type of replacement fluid) were at the discretion of the treating physician.

### Data collection

All data were extracted from the web-based electronic database of the PERFORM study (access through https://capctg.medbit.cn/), including baseline characteristics, daily SOFA score, daily laboratory test, daily triglyceride-lowering therapy, and follow-up data. The baseline characteristics included age, sex, body mass index (BMI), alcohol abuse, smoking, diabetes, time from abdominal pain onset to enrollment, disease severity scores on admission (Acute Physiology and Chronic Health Evaluation II, APACHE II score and SOFA score), presence of organ failure (respiratory, cardiovascular or renal), serum triglyceride level measured on admission.

### Outcome measures

The primary outcome was time to organ failure resolution within 14 days, which was defined as the number of days from enrollment to the last day the patient presented with any organ failure of respiratory, cardiovascular, or renal system. Patients who died within the first 14 days were considered to have unresolved OF and assigned the maximum OF duration of 14 days. Secondary outcomes include requirement of ICU admission, 28-day mortality, 60-day mortality, ICU-free days to day 14, and hospital length of stay (LOS).

### Statistical analysis

Continuous variables were presented as mean ± standard deviation (SD) or median and interquartile range (IQR) according to the normality of the data. Shapiro–Wilk test was used to check the normality of the distribution of variables. Comparison between groups was performed by Student’s t-test or Mann–Whitney U test as appropriate. Categorical variables were presented as frequency with percentage and compared using the chi-square test or Fisher exact test.

Kaplan–Meier curves were used to compare the cumulative incidence of organ failure resolution to 14 days after enrollment tested by log-rank test. Multivariable Cox proportional hazards regression models were employed to evaluate the association between plasmapheresis and organ failure resolution. Potential confounders were selected by a directed acyclic graph (DAG) (Additional file 1: Figure S1). DAG is a visual representation of potential causal relationships between variables connected by arrows [20]. The hypothesized relationships among variables were based on previous literature and expert experience [21]. A confounder is a common cause of both the exposure and the outcome, and a mediator is caused by the exposure and, in turn, causes the outcome [22]. A minimally sufficient adjustment set represents covariates such that the adjustment for this set of variables will minimize confounding bias when estimating the association between the exposure and the outcome [21, 23]. A minimally sufficient adjustment set [24] in the DAG was identified by DAGitty software, version 3.0 (www.dagitty.net). As a result, baseline triglyceride, APACHEII score, respiratory failure, renal failure, and cardiovascular failure were incorporated into the multivariable regression model. Hazard ratio (HR) and 95% confidence interval (CI) were calculated. The assumption of the proportional hazard was tested by checking the plots of Schoenfeld residuals over time. A subgroup analysis that controlled for baseline triglyceride has been performed to evaluate the effect of plasmapheresis on the primary outcome.

For secondary outcomes mentioned above, the comparisons between the two groups were carried out by the median regression model for skewed continuous data and the logistic regression model for categorical data. Covariates included in the model for secondary outcomes were the same as that for the primary outcome. Generalized estimating equation (GEE) model was applied to test differences in triglyceride-lowering effect across two groups.

Inverse probability of treatment weighting (IPTW) was performed as a sensitivity analysis to test the robustness of our results. The propensity score was estimated using multivariable logistic regression model including the same covariates selected by DAG. Group differences were compared by Wilcoxon rank-sum test for continuous variables and Chi-square test for binary variables weighted by the inverse probability of treatment. We also performed 1:1 propensity score matching (PSM) analysis, using the nearest neighbor method with the caliper width set to 0.1 of the standard deviations of the logit of the propensity score. Group differences in the PSM cohort were compared using Wilcoxon signed-rank test and McNemar's test for matched data. Time-to-event data were analyzed using the Kaplan–Meier method and were compared by the log-rank test.

A two-sided p value of less than 0.05 was considered as statistical significance. All statistical analyses were performed by SPSS version 26.0 (Chicago, IL, USA) and R version 4.2.3 (Alcatel-Lucent Bell Labs, New Jersey, USA).

## Results

### Study population

**Fig. 1:**
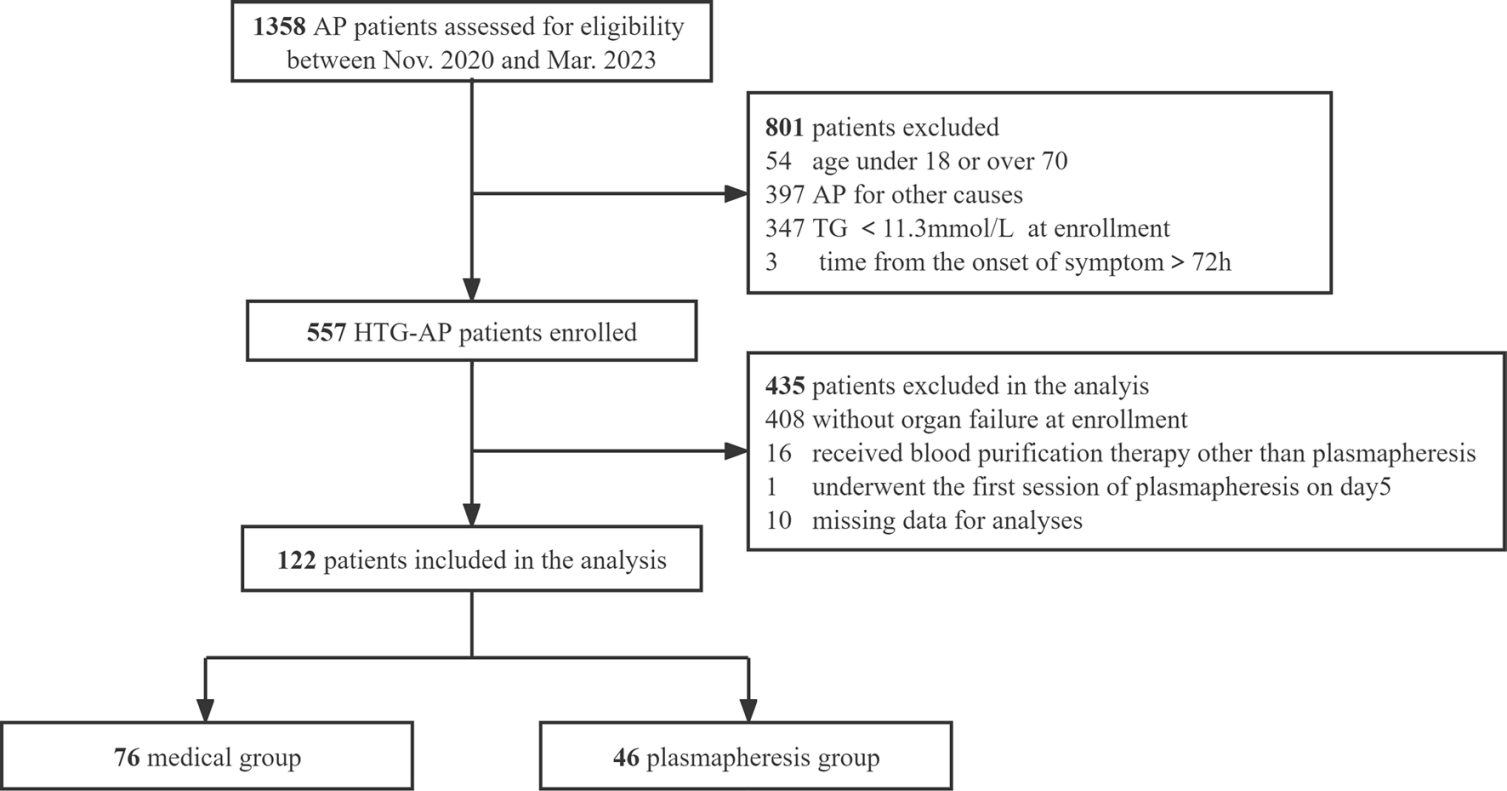
Flow chart

A total of 557 patients were enrolled in the PERFORM registry at the time of data extraction. After excluding patients without organ failure at enrollment (n = 408), receiving blood purification therapies other than plasmapheresis (n = 16), initiating plasmapheresis later than two days after enrollment (n = 1) and missing follow-up data for analyses (n = 10), 122 patients were included in the final analysis (Fig. 1).

The mean age of the study cohort was 39.47 years (standard deviation: 9.44). Overall, 78.7% (96/122) of the study patients were male, and most patients (91/122, 74.6%) were overweight or obese (Table 1). Most of the study patients were admitted to the hospital within 24 h from symptom onset (74/122, 60.7%). Respiratory failure (99/122, 81.1%) was the most common type of organ failure system. Patients undergoing plasmapheresis had higher baseline triglyceride levels (30.05 [18.20–61.95] vs. 20.87 [16.64–30.60] mmol/L, p = 0.007) and higher APACHEII scores (11.00 [8.00–15.25] vs. 8.50 [5.00–13.00], p = 0.012) than those receiving medical treatment.

**Table 1:** Baseline characteristics

Variable	All n = 122	Medical n = 76	Plasmapheresis n = 46	P value
Age, mean ± SD, y	39.47 ± 9.44	39.28 ± 10.05	39.78 ± 8.42	0.775
Male sex, n (%)	96 (78.7)	61 (80.3)	35 (76.1)	0.585
BMI, mean ± SD, kg/m^2^	27.73 ± 4.10	28.14 ± 4.30	27.04 ± 3.67	0.152
BMI categories, n (%)				0.155
18.5–24.9	31 (25.4)	15 (19.7)	16 (34.8)	
25.0–29.9	60 (49.2)	39 (51.3)	21 (45.7)	
≥ 30.0	31 (25.4)	22 (28.9)	9 (19.6)	
Smoking	48 (39.9)	26 (34.2)	22 (47.8)	0.136
Alcohol abuse	52 (42.6)	33 (43.4)	19 (41.3)	0.819
Diabetes, n (%)				0.545
Yes	23 (18.9)	13 (17.1)	10 (21.7)	
No	63 (51.6)	38 (50.0)	25 (54.3)	
Unknown	36 (29.5)	25 (32.9)	11 (23.9)	
Time interval^a^, n (%)				0.099
24 h	74 (60.7)	45 (59.2)	29 (63.0)	
48 h	37 (30.3)	21 (27.6)	16 (34.8)	
72 h	11 (9.0)	10 (13.2)	1 (2.2)	
APACHE II score, median(IQR)	10.00 (6.00–14.00)	8.50 (5.00–13.00)	11.00 (8.00–15.25)	0.012
APACHE II categories,n (%)				0.031
< 8	41 (33.6)	31 (40.8)	10 (21.7)	
≥ 8	81 (66.4)	45 (59.2)	36 (78.3)	
SOFA score, median(IQR)	3.00 (2.00–4.00)	3.00 (2.00–4.00)	3.50 (2.00–5.00)	0.398
*Organ failure*				
Respiratory, n (%)	99 (81.1)	59 (77.6)	40 (87.0)	0.202
Cardiovascular, n (%)	11 (9.0)	3 (3.9)	8 (17.4)	0.020
Renal, n (%)	34 (27.9)	21 (27.6)	13 (28.3)	0.940
Baseline triglyceride, median(IQR), mmol/L	23.71 (17.50–40.14)	20.87 (16.64–30.60)	30.05 (18.20–61.95)	0.007

BMI body mass index, *APACHE II* acute physiology and chronic health evaluation II; *SOFA* sequential organ failure assessment

^a^Time interval was categorized based on the time from the symptom onset to study enrollment

### Plasmapheresis

**Fig. 2:**
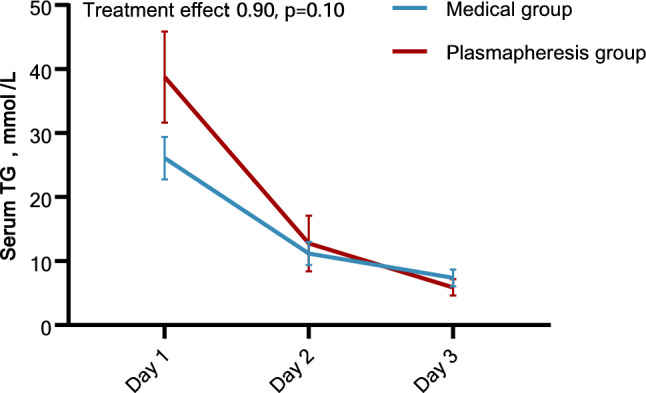
Generalized estimating equations of triglyceride levels between groups. The mean and 95% confidence interval (denoted by error bars) of triglyceride measures during the first three days of enrollment. Day1 was defined as the day of enrollment

Overall, 46 unique patients who underwent 65 plasmapheresis sessions were included in the plasmapheresis group (Additional file 1: Table S1). The majority of patients initiated the first session on the day of enrollment (35/46, 76.1%) and underwent only one session (31/46, 67.4%) during the index admission. Simple plasma exchange (41/46, 89.1%) was the most common type of plasmapheresis used in this cohort. Five patients (10.9%) underwent double filtration plasmapheresis (DFPP). The median volume of plasma used per procedure was 2000 ml, lasting 2.5 h. Analyses for dynamic changes of triglyceride within the first three days of admission showed no significant differences between groups (p = 0.10) (Fig. 2).

### Primary outcome

**Fig. 3:**
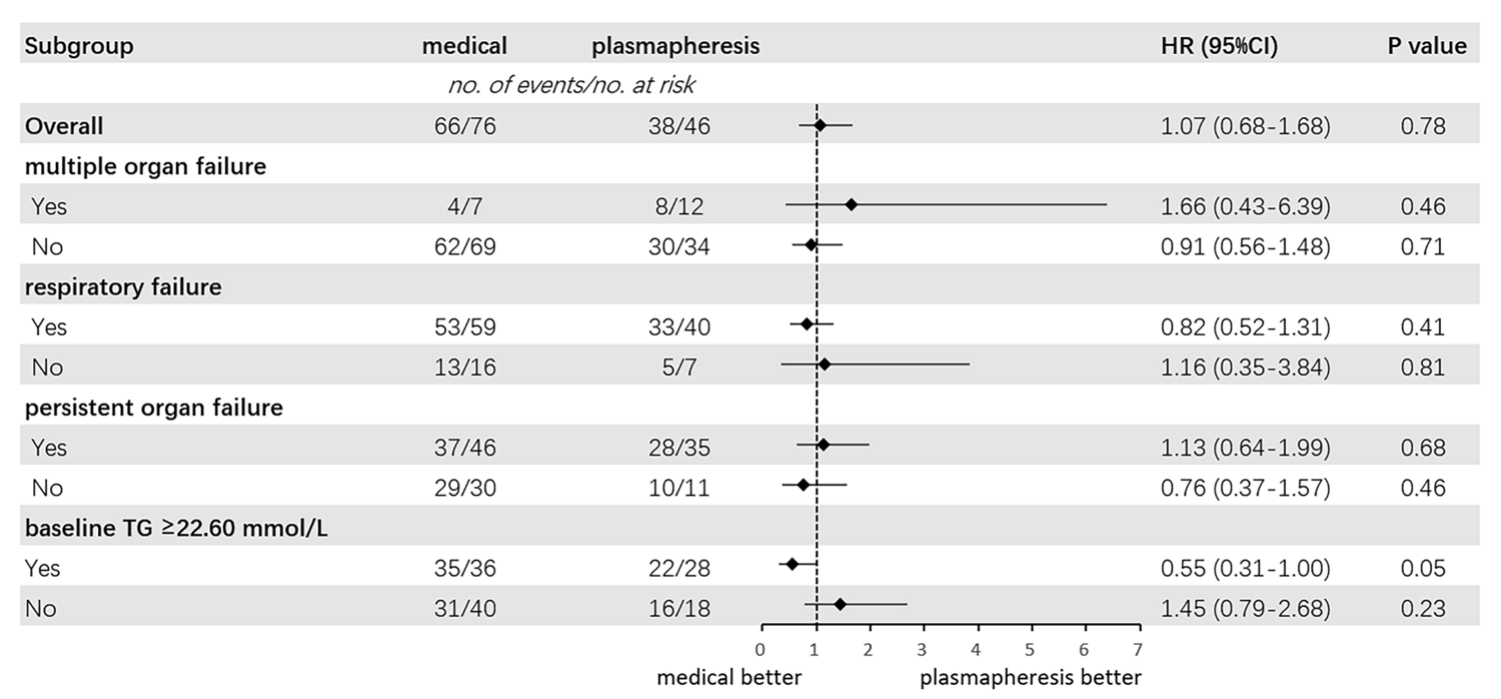
Subgroup analysis of plasmapheresis therapy on the incidence of organ failure resolution

There is no significant difference between groups for time to organ failure resolution within 14 days after enrollment (Log-Rank P = 0.60) (Additional file 1: Figure S2). In the multivariable Cox regression model, plasmapheresis therapy was not associated with accelerated organ failure resolution compared to medical treatment (HR = 1.07; 95%CI 0.68–1.68; P = 0.777) (Table 2). The subgroup analysis found no significant association between plasmapheresis and time to organ failure resolution across patients with either single or multiple organ failure. Also, the results were similar in patients with or without respiratory failure at enrollment (Fig. 3).

**Table 2:** Clinical outcomes

	Medical N = 76	Plasmapheresis N = 46	Effect estimate^a^ (95% CI)	P value
Primary outcome				
Time to organ failure resolution, d	4.00 (0.25–8.75)	4.00 (2.00–7.00)	HR, 1.07 (0.68 to 1.68)	0.777
Secondary outcomes				
Requirement of ICU admission	50 (65.8)	45 (97.8)	OR, 19.33 (2.20 to 169.81)	0.008
28-day mortality	2 (2.6)	2 (4.3)	OR, 0.67 (0.04 to 11.49)	0.785
60-day mortality	4 (5.3)	6 (13.0)	OR, 1.84 (0.35 to 9.61)	0.468
ICU-free days to day14, d	10.00 (2.25–14.00)	7.00 (2.00–10.00)	β, 0.85 (− 1.94 to 3.64)	0.547
Length of hospital stay, d	11.00 (7.00–18.75)	14.50 (9.75–20.25)	β, − 0.99 (− 5.58 to 3.61)	0.672

*ICU* intensive care unit, *HR* hazard ratio, *OR* odds ratio, *β* coefficient, *CI* confidence interval

^a^Cox regression model or Logistic regression model or Median regression model with the adjustment of APACHEII, Baseline triglyceride, respiratory failure, cardiovascular failure, and renal failure

### Secondary outcomes

After multivariable adjustment, there were no significant differences in ICU-free days to day 14, length of hospital stay, 28-day mortality, or 60-day mortality between groups. However, the plasmapheresis group had significantly higher ICU requirements (97.8% [45/46] vs. 65.8% [50/76]; OR, 19.33; 95%CI 2.20 to 169.81; P = 0.008) compared to the medical group (Table 2).

### Sensitivity analysis

IPTW analyses yielded similar results as no significant association was found between plasmapheresis and organ failure resolution (median (IQR), 4.00 (1.00–10.23) vs. 4.00 (2.00–6.00); P = 0.584) (Additional file 1: Table S2). Kaplan–Meier curves also showed no difference in the cumulative incidence of organ failure resolution within 14 days between the two groups (Log-Rank P = 0.60) (Additional file 1: Figure S3). For secondary outcomes, the plasmapheresis group had fewer ICU-free days to day14 (median (IQR), 7.00 (3.00–10.00) vs. 9.14 (2.01–14.00); P = 0.003) compared to the medical group. Other secondary outcomes showed the similar results as primary analyses. Additionally, the PSM analysis also demonstrated that there was no significant difference between groups for the primary outcome. (Additional file 1: Table S2, Figure S4).

## Discussion

In this study, we found that early plasmapheresis was not associated with accelerated organ failure resolution compared to exclusive medical treatment in HTG-AP patients with early organ failure. Furthermore, the use of plasmapheresis was associated with a higher rate of ICU admission compared to the medical treatment.

International consensus suggests that the presence and duration of organ failure is the key determinant of disease severity in acute pancreatitis [25]. Garg et al. recommended that severe HTG-AP patients with organ failure should be offered intensive care and initiate plasmapheresis therapy as soon as possible to lower triglyceride with a target of < 5.65 mmol/L [26]. However, there is a paucity of high-quality evidence justifying the use of plasmapheresis in this population. In a retrospective study conducted by Lu et al. [27], they found that DFPP was associated with a rapid reduction of triglyceride but not associated with reduced incidence of persistent OF when compared to medical treatment. Taken together, there is a clinical equipoise on whether plasmapheresis should be universally applied in severe HTG-AP patients.

Of note, Gubensek [28] proposed that DFPP may not be technically feasible to improve organ (mainly respiratory) failure since relatively small sized molecules like free fatty acids (FFA) and inflammatory cytokines could not be removed by DFPP. In the pathophysiology of HTG-AP, the lipotoxicity of FFA generated from triglyceride lipolysis is critical in the development of organ failure [29, 30]. In this regard, conventional plasma exchange might be more effective as it removes not only triglyceride but also FFA. However, Jin et al. [31] showed no significant differences in the lowering tendency of APACHE II score during hospitalization, the incidence of persistent OF, or organ support between plasma exchange and medical treatment. In our study, 89.1% (41/46) patients underwent plasma exchange and the results showed plasmapheresis was not associated with more rapid organ failure resolution, even in subgroup patients with multiple organ failure or respiratory failure, indicating that FFA removal may not be the main reason why plasmapheresis (either plasma exchange or DFPP) could not improve organ failure.

One possible explanation is that the changes in the amount of triglyceride or FFA removed by plasmapheresis are not necessarily result in clinical improvement [32]. Once the cascade effect of systemic inflammatory response was triggered by hypertriglyceridemia, it may self-perpetuate even if the triglyceride level in circulation dropped [17, 27, 33]. Another is that plasmapheresis was not associated with a more efficient reduction of triglyceride compared to medical treatment, as our results showed, which is also supported by several previous studies [31, 34, 35].

To the best of our knowledge, this is the first large-scale study exploring whether the use of plasmapheresis was associated with rapid organ failure resolution in a cohort of severe HTG-AP patients with early organ failure. It is important to note that there are some limitations in this study. First, the study was flawed by its observational nature, precluding the possibility of causal relationship analyses. Second, the prescriptions for plasmapheresis were not standardized across the centers. Previous studies had shown that plasmapheresis type [28], anticoagulation [16], and replacement fluid [32] were associated with different effects for HTG-AP patients. Third, since serum triglyceride levels tend to decrease markedly after 24 h of fasting in the majority of patients [36, 37], it is possible that we did not capture the highest triglyceride level during each episode as we recruited patients within 72 h of symptom onset. This may explain why the baseline triglyceride levels were not as high as those in previous small retrospective studies [38, 39] and case series [40, 41]. Fourth, there was a low prevalence of multiple organ failure (15.6%, 19/122), and in particular, a low prevalence of shock (9.0%, 11/122), resulting in a low 28-day mortality rate of 3.3%. The absence of benefit of plasmapheresis among patients with more severe acute pancreatitis and multiple organ failure is still unclear, even if subgroup analyses have been performed.

## Conclusions

In this large multicenter observational study, we found that early plasmapheresis therapy, compared with exclusive medical treatment, was not associated with more rapid organ failure resolution in HTG-AP patients with early organ failure. Further well-designed prospective randomized controlled trials are needed to confirm our findings.

### Supplementary Information

**Additional file 1: Table S1.** Description of plasmapheresis therapy. **Table S2.** Clinical outcomes after IPTW or PSM. **Figure S1.** Directed acyclic graph for time to organ failure resolution. **Figure S2.** Time to organ failure resolution within 14 days in the primary analysis. **Figure S3.** Time to organ failure resolution within 14 days in the IPTW analysis. **Figure S4.** Time to organ failure resolution within 14 days in the PSM analysis.

## Data Availability

The datasets analysed during the current study are available from the corresponding author on reasonable request.

## Abbreviations

HTG-AP: Hypertriglyceridemia-associated acute pancreatitis
SOFA: Sequential organ failure assessment
APACHE II: Acute physiology and chronic health evaluation II
ICU: Intensive care units
OF: Organ failure
BMI: Body mass index
LOS: Length of stay
SD: Standard deviation
IQR: Interquartile range
DAG: Directed acyclic graph
HR: Hazard ratio
CI: Confidence interval
GEE: Generalized estimating equation
IPTW: Inverse probability of treatment weighting
DFPP: Double filtration plasmapheresis
FFA: Free fatty acids
