# Supplementary material for: Impact of therapeutic plasmapheresis on the duration of organ failure in patients with hypertriglyceridemia-associated acute pancreatitis
Source: Ann Intensive Care. 2024 Apr 15;14:57. doi: 10.1186/s13613-024-01285-3 (PMC11018571; doi:10.1186/s13613-024-01285-3)

Table S1. Description of plasmapheresis therapy.

| Number of plasmapheresis |  |
| --- | --- |
| 1 session | 31 (67.4%) |
| 2 sessions | 13 (28.3%) |
| 4 sessions | 2 (4.3%) |
| The first plasmapheresis |  |
| Day 1 | 35 (76.1%) |
| Day 2 | 11 (23.9%) |
| Method of plasmapheresis |  |
| Simple plasma exchange | 41 (89.1%) |
| Double filtration plasmapheresis | 5 (10.9%) |
| Plasma volume per procedure, ml | 2000 (2000-3000) |
| Time per procedure, h | 2.5 (2.0-3.4) |

Data were presented as n (%) or medians (IQRs).

Table S2. Clinical outcomes after IPTW or PSM.

|  | After IPTW | | | After PSM | | |
| --- | --- | --- | --- | --- | --- | --- |
|  | Medical  N=118 | Plasmapheresis  N=122 | P value | Medical  N=31 | Plasmapheresis  N=31 | P value |
| ***Primary outcome*** |  |  |  |  |  |  |
| Time to organ failure resolution, d | 4.00 (1.00-10.23) | 4.00 (2.00-6.00) | 0.584 | 7.00 (3.50-12.50) | 4.00 (2.00-6.00) | 0.088 |
| ***Secondary outcomes*** |  |  |  |  |  |  |
| Requirement of ICU admission | 81 (68.6) | 117 (95.9) | <0.001 | 20 (64.5) | 30 (96.8) | 0.002 |
| 28-day mortality | 3 (2.5) | 3 (2.4) | 1.00 | 2 (6.5) | 1 (3.2) | 1.00 |
| 60-day mortality | 7 (5.9) | 10 (8.2) | 0.494 | 3 (9.7) | 3 (9.7) | 1.00 |
| ICU-free days to day14, d | 9.14 (2.01-14.00) | 7.00 (3.00-10.00) | 0.003 | 9.00 (1.50-14.00) | 7.00 (3.00-10.00) | 0.354 |
| Length of hospital stay, d | 11.00 (7.00-20.87) | 14.00 (10.38-18.00) | 0.156 | 11.00 (8.00-20.00) | 15.00 (10.00-17.50) | 0.837 |

Figure S1. Directed acyclic graph for time to organ failure resolution.


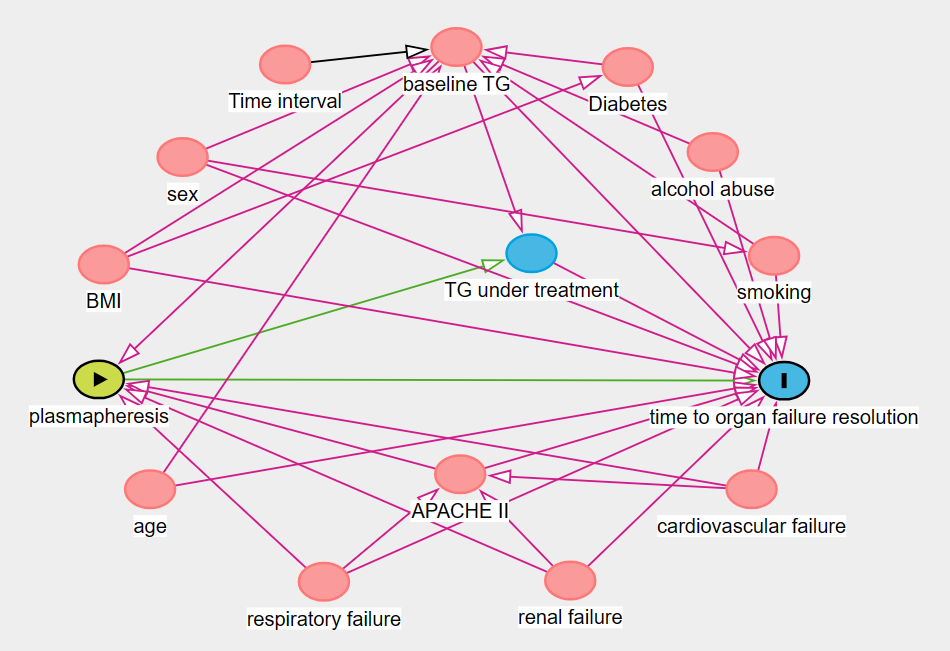


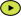
 exposure


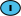
 outcome


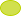
 ancestor of exposure


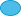
 ancestor of outcome


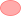
 ancestor of exposure and outcome


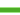
 causal path


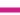
 biasing path

Abbreviations:BMI, body mass index; TG, triglyceride; APACHE II, acute physiology and chronic health evaluation II

The DAG algorithm identified the following minimal sufficient adjustment set: baseline TG, APACHE II, respiratory failure, renal failure, and cardiovascular failure.

Figure S2. Time to organ failure resolution within 14 days in the primary analysis.


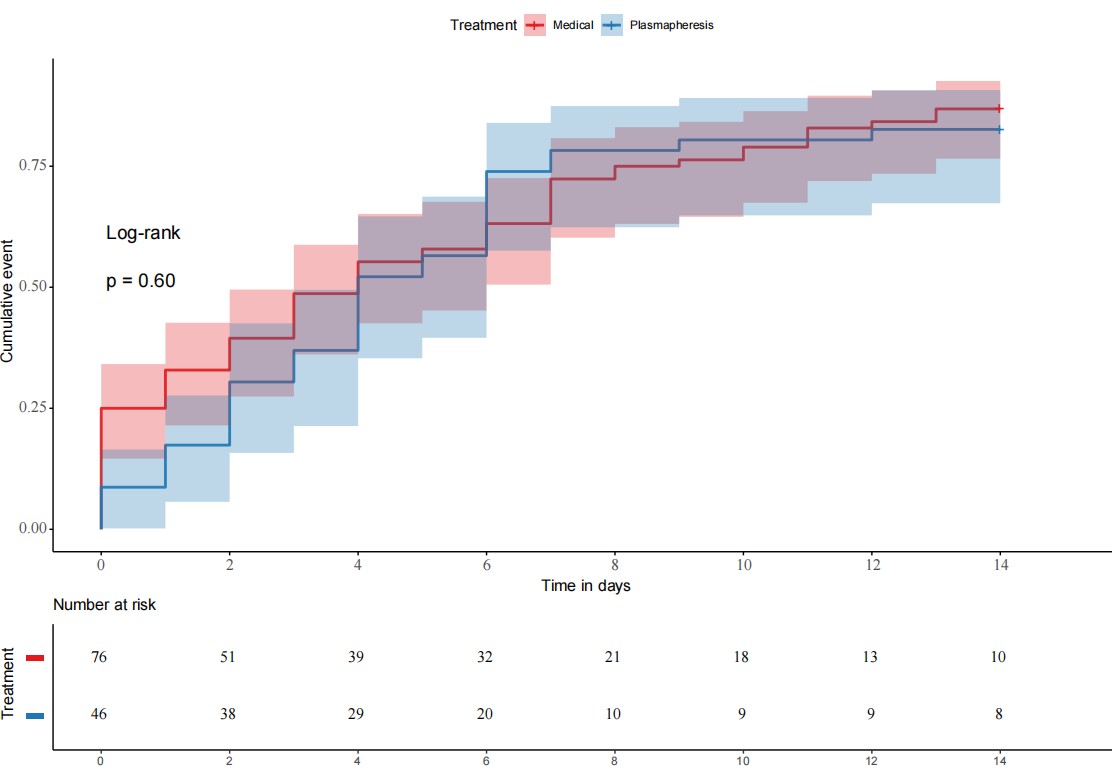


Figure S3. Time to organ failure resolution within 14 days in the IPTW analysis.


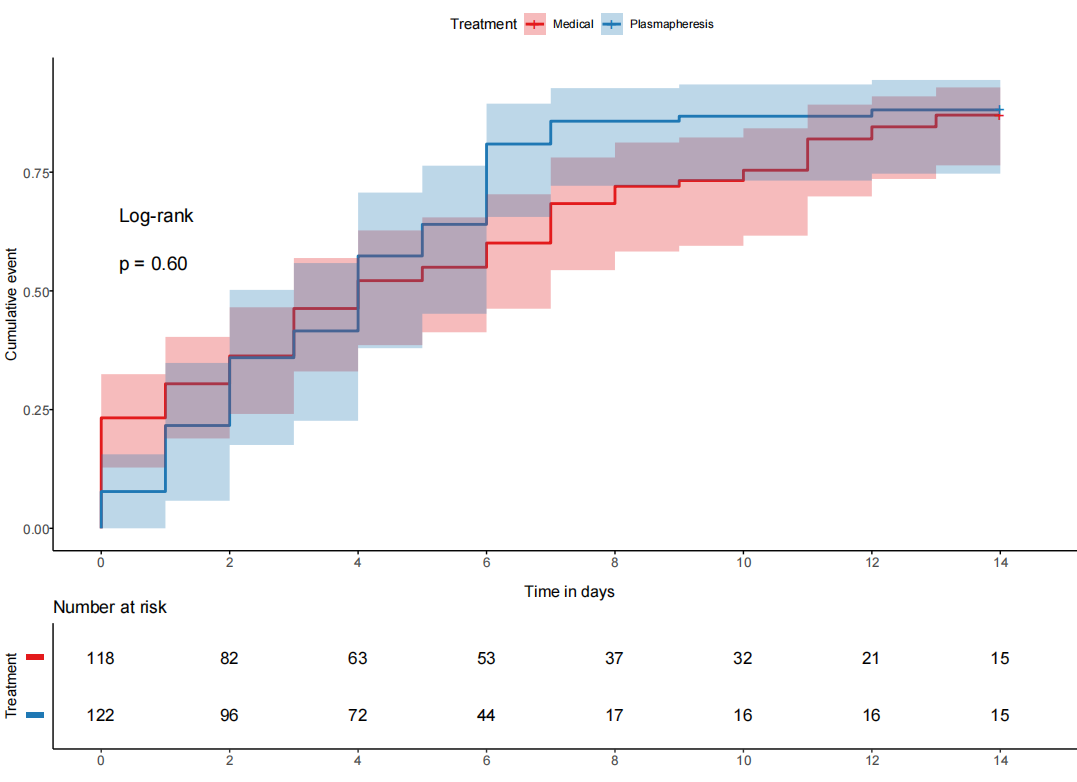


Figure S4. Time to organ failure resolution within 14 days in the PSM analysis.


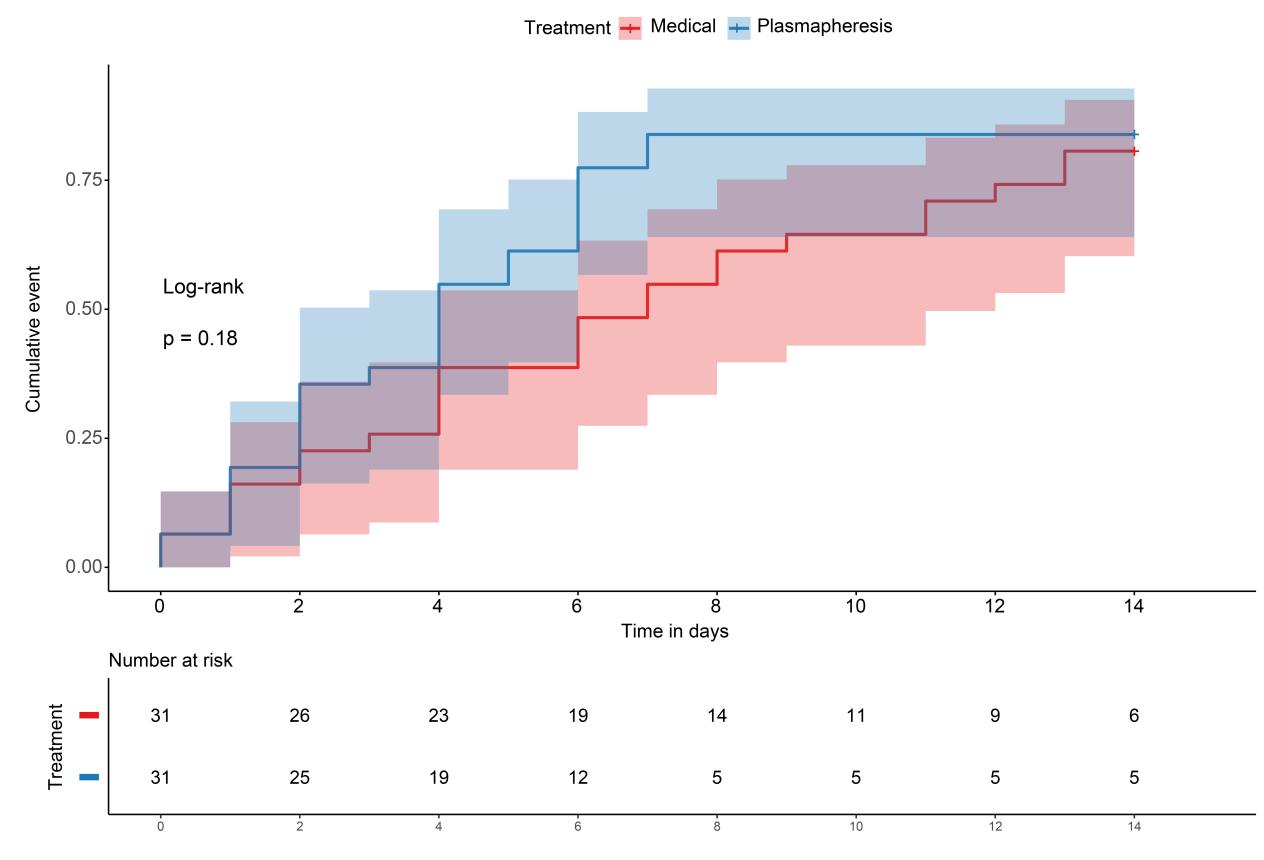

Supplement: Supplementary file 1 — Additional file 1: Table S1. Description of plasmapheresis therapy. Table S2. Clinical outcomes after IPTW or PSM. Figure S1. Directed acyclic graph for time to organ failure resolution. Figure S2. Time to organ failure resolution within 14 days in the primary analysis. Figure S3. Time to organ failure resolution within 14 days in the IPTW analysis. Figure S4. Time to organ failure resolution within 14 days in the PSM analysis. [file 13613_2024_1285_MOESM1_ESM.docx]
